# Keynote 4

**DOI:** 10.1093/eurpub/ckac092.004

**Published:** 2022-08-29

## Abstract

**Title of HEPA 2022 Keynote presentation:**

Physical activity as a key factor for cross-cutting risk assessment.

**Description of HEPA 2022 Keynote presentation:**

Moving little, sleeping badly or eating poorly, ignoring our biological rhythms are all major risks factors for our health. Physical activity has long been mainly regarded as a factor of health improvement, whereas it is the lack of activity which constitutes a hazard for human health. Actually, physical (in)activity-related factors (PAFs) – as both physical inactivity and sedentarity—must be intrinsically analysed as a health risk. In everyday language, “risk” and “hazard” are often used confusedly when referring to a threat. However, these two words refer to two distinct concepts, and at the earliest stage of assessment, PAFs have to be characterized as a hazard. The usual approach in toxicology leads to set health reference values. In view of these values, the calculation of exposure therefore makes possible to quantify the risk. In addition, because of the way that PAFs interfer with exposure-related physiopathogical response, they have to be taken into account in multifactorial risk-assessments. Should we not consider the individual as a whole, in a close relationship to its environment and move away from silo-based approaches towards an integrated vision of health risk? This assumption calls for due consideration to be given to physical activity in the assessment of human exposure-related to health risks. The talk will aim to demonstrate the need for an integrated and cross-cutting risk assessment based on scientific evidence in which physical (in)activity can now definitely take its place. At last, isn’t it a way to simply, concretely, illustrate the conceptual approach of exposome?

